# Root and leaf metabolite profiles analysis reveals the adaptive strategies to low potassium stress in barley

**DOI:** 10.1186/s12870-018-1404-4

**Published:** 2018-09-10

**Authors:** Jianbin Zeng, Xiaoyan Quan, Xiaoyan He, Shengguan Cai, Zhilan Ye, Guang Chen, Guoping Zhang

**Affiliations:** Agronomy Department, Institute of Crop Science, Zhejiang University, Hangzhou, 310058 China

**Keywords:** Barley, Metabolome, Pathway, Genotypes, Low potassium tolerance

## Abstract

**Background:**

Potassium (K) deficiency in arable land is one of the most important factors affecting crop productivity. Development of low K (LK) tolerant crop cultivars is regarded as a best economic and effective approach for solving the issue of LK. In previous studies, we found a wider variation of LK tolerance in the Tibetan wild barley accessions than cultivated barley. However, the mechanism of LK tolerance in wild barley is still elusive.

**Results:**

In this study, two wild barley genotypes (XZ153, LK tolerant and XZ141, LK sensitive) and one cultivar (LuDaoMai, LK tolerant) was used to investigate metabolome changes in response to LK stress. Totally 57 kinds of metabolites were identified in roots and leaves of three genotypes at 16 d after LK treatment. In general, accumulation of amino acids and sugars was enhanced in both roots and leaves, while organic acids were reduced under LK stress compared to the control. Meanwhile, the concentrations of the negatively charged amino acids (Asp and Glu) and most organic acids was reduced in both roots and leaves, but more positively charged amino acids (Lys and Gln) were increased in three genotypes under LK. XZ153 had less reduction than other two genotypes in biomass and chlorophyll content under LK stress and showed greater antioxidant capacity as reflected by more synthesis of active oxygen scavengers. Higher LK tolerance of XZ153 may also be attributed to its less carbohydrate consumption and more storage of glucose and other sugars, thus providing more energy for plant growth under LK stress. Moreover, phenylpropanoid metabolic pathway mediated by PAL differed among three genotypes, which is closely associated with the genotypic difference in LK tolerance.

**Conclusions:**

LK tolerance in the wild barley is attributed to more active phenylpropanoid metabolic pathway mediated by PAL, energy use economy by reducing carbohydrate consumption and storage of glucose and other sugars, and higher antioxidant defense ability under LK stress.

**Electronic supplementary material:**

The online version of this article (10.1186/s12870-018-1404-4) contains supplementary material, which is available to authorized users.

## Background

Potassium (K) is one of the essential macronutrients for plant growth and development [1, 2]. As one of the most abundant cations in living plant tissues, K plays a crucial role in many biophysical and biochemical processes including enzyme activation, ion homeostasis, osmoregulation, protein synthesis, etc. [3, 4]. Although K is abundant in the earth crust, its available forms for plant uptake, mainly ionic and exchangeable K is low in the most arable lands [5]. Moreover, available K level in most of the soils is being reduced gradually due to the inadequate K recycling during crop production. In short, K deficiency in arable land has become a major restricting factor for sustainable crop production in most areas of the world [6].

On the other hand, there is a large difference among plant species and genotypes within a species in the tolerance to K deficiency [7, 8]. Considerable variation in efficiency of K uptake and utilization has been reported among genotypes for all the major crop species, indicating that the low K tolerance is genetically controlled and can be improved through genetic manipulation [6]. However, little progress was made in the development of low K tolerant crop cultivars. One of the major limitations is little understanding of the mechanisms for the observed genotypic difference in low K tolerance [8]. Accordingly, it is imperative to reveal the relevant mechanisms by using new methods and approaches.

Barley (*Hordeum vulgare* L.) is the fourth most important cereal crop in terms of planting area in the world, only after wheat, maize and rice. Although barley shows the greater tolerance to low nutrients, its growth and yield will be greatly inhibited when K supply is not sufficient, and this issue is more particular for the modern high-yield cultivars [9]. Thus, it is important to improve the efficiency of K uptake and utilization in barley. However, narrow genetic diversity in the cultivated barley has become a bottleneck for further genetic improvement [10]. On the other hand, Tibetan annual wild barley, as one of the ancestors of cultivated barley [11], is rich in genetic diversity and the elite accessions tolerant to abiotic stresses, such as drought and salinity [12–14], as well as to poor fertility, including low K [15–17]. However, the physiological and molecular mechanisms conferring low K tolerance in wild barley remain unclear.

Currently, metabolomics has been widely used as a powerful tool for analyzing a large number of compounds from a given plant species at a certain developmental stage or under particular environmental conditions, providing a broad view of systematic adjustment in metabolic processes [18, 19]. Actually, many metabolomics studies have been conducted to understand the mechanisms of abiotic stress tolerance in plants, including drought [20], salinity [21], combined stress of drought and high temperature [22] and cadmium toxicity [23]. Meanwhile, metabolomics analysis have also been used in revealing the mechanisms of low nutrient tolerance in higher plants, such as the responses of maize and barley to low nitrogen stress [24, 25], of common beans and barley to P deficiency [26, 27] and of *Arabidopsis* to low sulfur level [28]. Moreover, metabolite profiles were also reported in *Arabidopsis* and tomato plants exposed to normal and low-K conditions [29, 30]. However, little information is available for the metabolite difference among the plant genotypes differing in low K tolerance in their responses to low-K stress.

In the present study, one cultivated (LuDaoMai) and two wild (XZ153 and XZ141) barley genotypes differing in low K tolerance were used according to the previous study [16], to compare their metabolic changes in both roots and leaves responding to low K stress by using gas chromatography-mass spectrometry (GC-MS) method. The primary aim of this study was to identify the compounds associated with the low K tolerance among these genotypes and understand the mechanisms underlying low K tolerance in wild and cultivated barley.

## Results

### The influence of LK stress on plant growth of Tibetan wild barley and cultivated barley

**Fig. 1:**
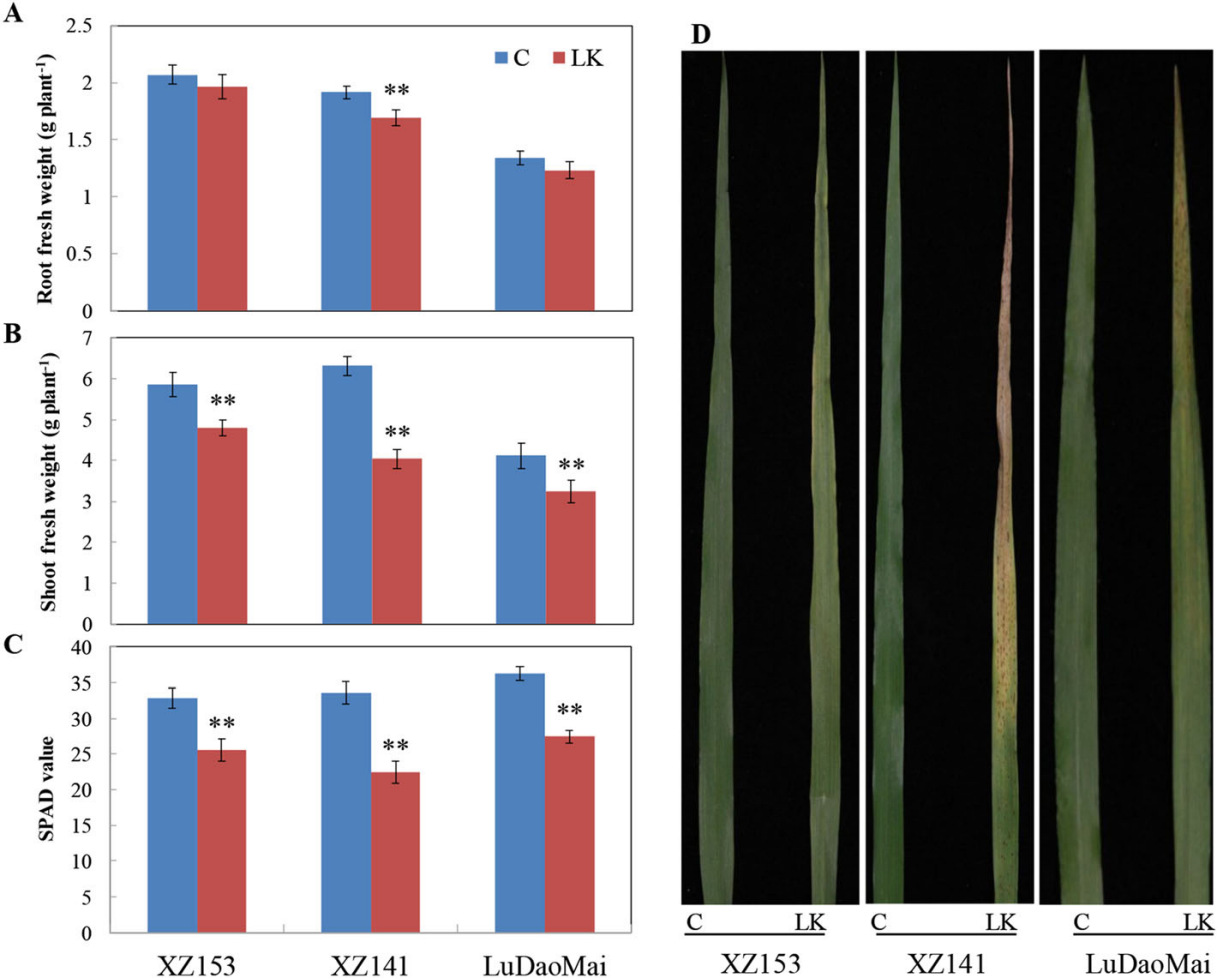
Growth performances of the three barley genotypes under control (C) and low potassium (LK) conditions. **a** Root fresh weight (*n* = 4); (**b**) Shoot fresh weight (*n* = 4); (**c**) SPAD value (chlorophyll content) (*n* = 6); (**d**) Symptoms of LK from old leaves. * and ** indicate significant (*p* < 0.05) and highly significant (*p* < 0.01) differences between control treatment, respectively

Three barley genotypes used in this experiment showed a distinct difference in LK tolerance in terms of biomass after exposure to LK stress for 16 days (Fig. 1a and b). The reduction of root fresh weight (RFW) and shoot fresh weight (SFW) caused by LK in comparison with control was approximately 8% and 25%, respectively, averaged over three genotypes (Fig. 1a and b). The reduced extent differed among three genotypes, with XZ141 showing a significant reduction in RFW and the reduction of other two genotypes was not significant (Fig. 1a). Although SFW was significantly reduced for all three genotypes in the LK-treated plants, XZ153 showed obviously less reduction than other two genotypes (Fig. 1b). Similarly, SPAD values (chlorophyll content) of all three genotypes were significantly reduced under LK stress, with XZ153 being least (about 22%) and XZ141 being most (about 33%) reduced, respectively (Fig. 1c). Moreover, obvious symptoms of K deficiency could be observed in the old leaves of XZ141, while LuDaoMai and XZ153 showed the moderate and slight symptoms, respectively (Fig. 1d). In short, the current results confirmed the previous findings that XZ153 were more LK-tolerant than XZ141 and LuDaoMai.

### The changes in metabolite profiles of three genotypes in response to LK stress

**Fig. 2:**
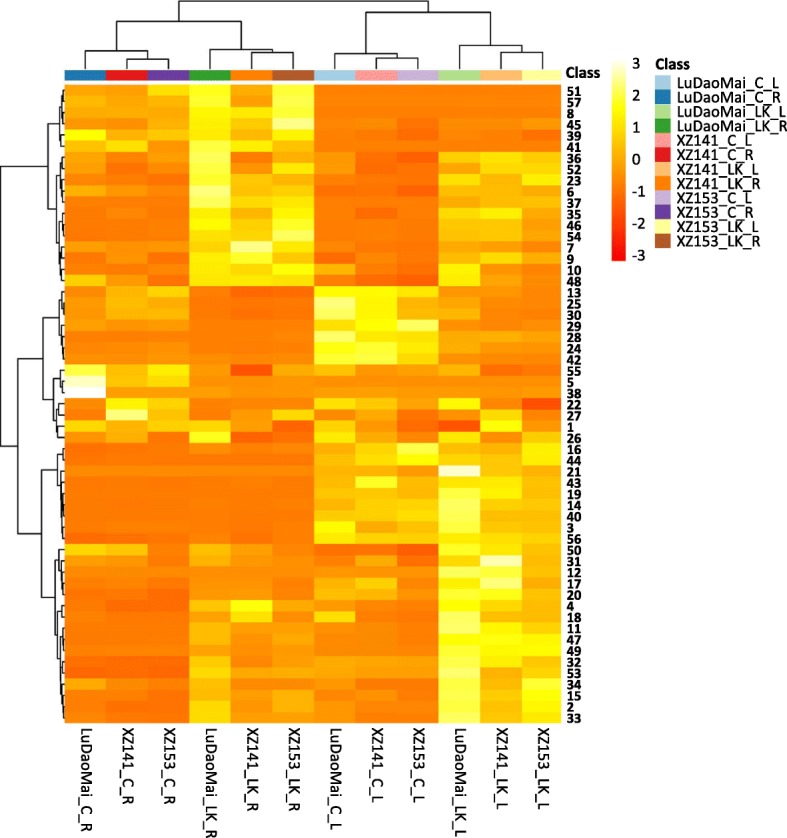
Heatmap and hierarchical cluster analysis for the 57 detected metabolites in the three barley genotypes. C: control; LK: low potassium; R: root; L: leaf. The names of the metabolites shown in the corresponding numbers from 1 to 57 were listed in Additional file 1 Table S1

Totally 57 kinds of metabolites were detected, which changed significantly under LK stress relative to control (C) in both roots and leaves of three genotypes (Additional file 1: Table S1). In order to identify the different metabolites between Tibetan wild and cultivated barley in response to K nutrition, all metabolite profiles, consisting of 72 barley samples (i.e. consisting of two tissues, three genotypes, two K levels and six replicates), were performed by Heatmap and hierarchical cluster analysis. According to Heatmap analysis (Fig. 2), an obvious separation could be observed between two plant tissues within the control or LK treatment, with 72 samples being clearly grouped into two classes, every 36 samples for root and leaf, respectively. Furthermore, two subclasses, consisting of the samples from control and LK treatment respectively, could be divided within the leaf and root samples (Fig. 2).

### Metabolite profiles and pathway in barley roots responding to LK stress

**Fig. 3:**
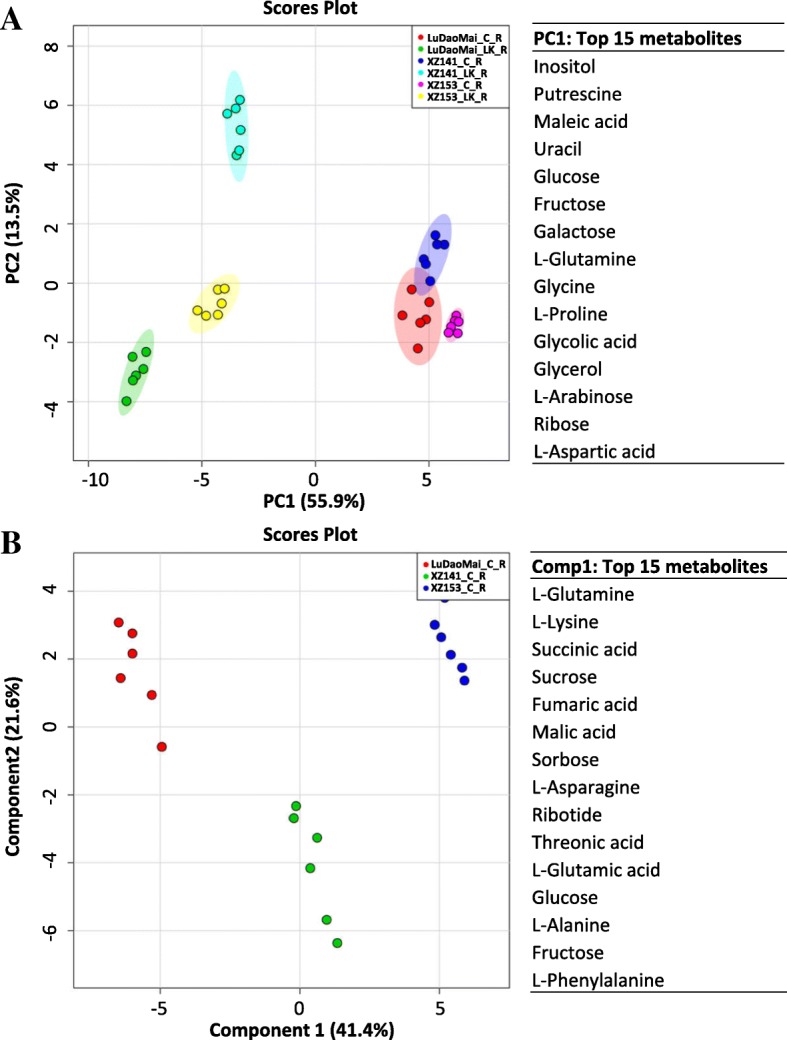
Root metabolome variation analysis and top 15 metabolites for the PC1 and Comp 1. **a** Principal component analysis (PCA) of root metabolome variation among samples and the top 15 metabolites contributing to the PC1; (**b**) Partial least squares discriminant analysis (PLS-DA) of root metabolome variation among samples. PC1: the first principal component; PC2: the second principal component; Comp1: component 1; C: control; LK: low potassium; R: root (*n* = 6)

Totally 55 metabolites in roots were changed significantly in their concentrations under LK stress compared to the control, including 18 amino acids, 14 sugars and polyols, 16 organic acids and 7 other compounds (Table 1). In order to reveal the effect of LK stress on metabolite alternation, PCA analysis was conducted on those 55 metabolites (Fig. 3 and Additional file 2: Table S2). Obviously, the samples of both control and LK treatment could be clearly separated by PC1, accounting for approximately 55.9% of the total variation (Fig. 3a). The main metabolites contributing to the PC1 included inositol, putrescine, maleic acid, L-glutamine, glucose and other 10 metabolites (Fig. 3a). The PC2 could clearly separate the LK-treated samples within three genotypes but did not separate the control samples (Fig. 3a). Thus, partial least squares-discriminant analysis (PLS-DA) was further conducted to determine the difference (Fig. 3b and Additional file 3: Table S3). Under the control condition, the metabolites in roots contributing to the component 1 (accounting for approximately 41.4%) were L-glutamine, L-lysine, succinic acid, sucrose, fumaric acid and other 10 metabolites (Fig. 3b and Additional file 3: Table S3).

**Table 1:** Fold changes of detected metabolites in roots of the three barley genotypes under LK stress

Metabolite name	Log_2_(LK/C)^a^			Metabolite name	Log_2_(LK/C)^a^		
	XZ153	XZ141	LuDaoMai		XZ153	XZ141	LuDaoMai
**Amino acid** ^b^				**Sugars and polyols**			
L-Alanine	−0.1	− 0.53**	0.11	Maltose	−0.50**	−1.42**	− 0.93**
Valine	1.76**	2.85**	1.55**	Sucrose	0.47**	0.09	1.31**
L-Leucine	1.58**	2.30**	1.07**	Inositol	2.19**	1.69**	2.64**
L-Isoleucine	1.56**	1.36**	1.73**	Trehalose	1.52**	−0.32	1.33**
L-Proline	1.78**	1.76**	1.78**	**Organic acid**			
Glycine	0.91**	1.03**	1.45**	Glycolic acid	1.98**	1.41**	1.94**
L-Serine	0.43**	0.55**	0.74**	Benzoic acid	1.28**	0.78**	1.18**
L-Threonine	0.86**	0.82**	0.91**	Maleic acid	3.71**	3.52**	4.55**
β-Alanine	−0.61**	−0.80**	−0.1	Succinic acid	−2.08**	−1.99**	−0.36**
L-Aspartic acid	−2.94**	−3.56**	−2.21**	Glyceric acid	2.17**	1.52**	2.02**
GABA	0.12	−0.82**	0.01	Fumaric acid	0.59**	0.57**	1.75**
L-Glutamic acid	−2.42**	−3.00**	−1.25**	Malic acid	−1.39**	−2.07**	−0.73**
L-Phenylalanine	0.59**	0.04	0.39**	Ketoglutaric acid	−4.0**	−5.30**	−4.47**
Ornithine	1.55**	0.24*	1.13**	Threonic acid	0.50*	0.67	1.54**
L-Lysine	2.40**	1.16**	0.28*	Ribotide	−1.00**	−1.53**	−3.72**
Tyrosine	0.05	−0.54**	−0.26	Shikimic acid	−0.15	− 0.06	1.07**
L-Asparagine	0.85**	−0.12	0.52**	Citric acid	−1.12**	−2.0**	−0.55**
L-Glutamine	3.55**	2.48**	2.60**	Quinic acid	0.08**	−0.35**	0.21*
**Sugars and polyols**				Ascorbic acid	0.42**	0.35*	0.62**
Xylose	2.95**	2.14**	3.00**	Hexadecanoic acid	0.99**	0.50*	1.02**
L-Arabinose	1.31**	1.11**	1.59**	Hexanoic acid	−0.56**	−0.11	0
Ribose	1.70**	0.82**	1.67**	**Others**			
Xylitol	0.1	−0.05	0.50**	Urea	−3.68**	−5.74**	−6.00**
Glycerol	1.76**	1.17**	1.43**	Mannitol	0.63**	0.45	1.87**
2-Keto-L-gluconic acid	0.45**	0.13	−0.18*	Putrescine	5.28**	5.34**	5.35**
Fructose	3.84**	3.78**	4.56**	Uracil	2.98**	1.35**	1.95**
Galactose	2.82**	2.13**	3.55**	Pipecolinic acid	2.43**	4.33**	1.87**
Glucose	2.90**	2.56**	4.07**	Pyroglutamic acid	−0.06	−0.94**	0.92**
Sorbose	3.64**	3.36**	3.97**	O-Methyl-Inositol	1.15**	0.91**	1.14**

^a^The fold changes were calculated using the formula log_2_ (LK/C), *LK* Low potassium, *C* control. ^b^The bolder parts of the table are the different categories of metabolites.* and ** indicate significant (*p* < 0.05) and highly significant difference (*p* < 0.01), respectively

**Fig. 4:**
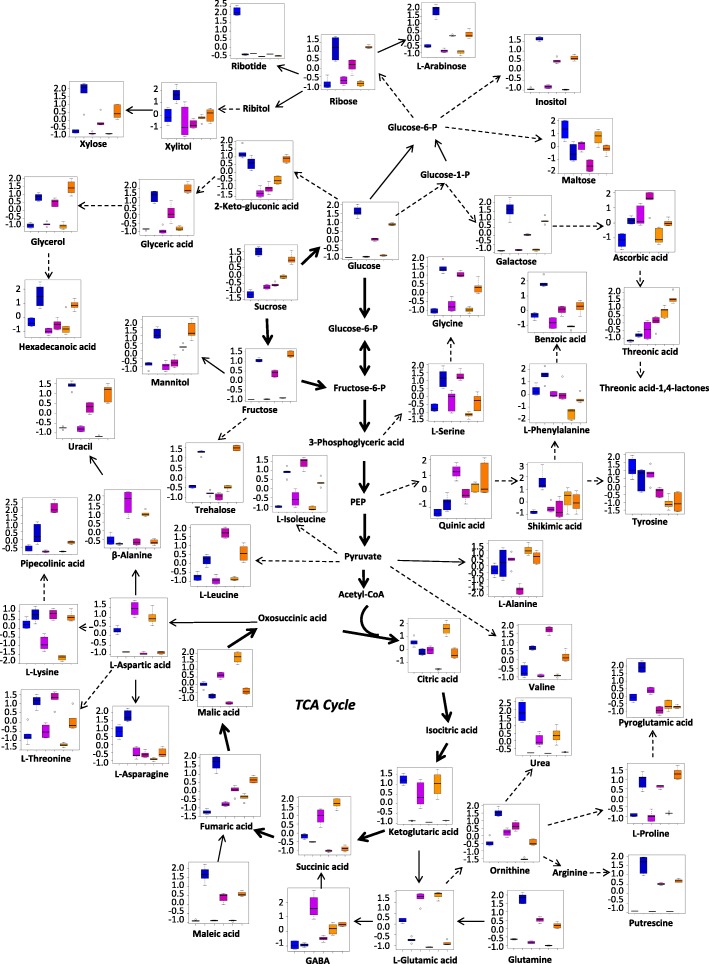
Metabolic pathways in roots of the three barley genotypes in response to low-K stress. The six columns from left to right on the X-axis represent LuDaoMai (control and treatment), XZ141 (control and treatment) and XZ153 (control and treatment), respectively. The concentration of each metabolite on the Y-axis is presented after normalized on Metaboanalyst software (http://www.metaboanalyst.ca/)

Under LK stress, 38 and 11 metabolites in roots of XZ153, 30 and 16 metabolites in roots of XZ141, and 40 and 10 metabolites in roots of LuDaoMai showed significant up-accumulation and down-accumulation, respectively (Table 1). Overall, LK stress caused a dramatic increase of several basic or neutral amino acids (Fig. 4 and Table 1). XZ153 accumulated higher contents of L-phenylalanine (approximately 1.5-fold), ornithine (approximately 2.9-fold), L-lysine (approximately 5.3-fold), L-asparagine (approximately 1.8-fold) and L-glutamine acids (approximately 11.7-fold) than XZ141 and LuDaoMai (Table 1). While acidic amino acids, such as aspartic acid and glutamate, decreased in all three genotypes under LK stress, with XZ141 showing the largest reduction (Table 1). The inhibition of aspartic acid synthesis leads to a reduction of downstream β-alanine (Fig. 4). In addition, L-alanine and tyrosine were obviously reduced in XZ141, but not in the other two genotypes (Table 1).

Accumulation of most sugars and their related metabolites were enhanced under LK stress (Fig. 4 and Table 1). The contents of downstream metabolites, such as fructose, glucose, mannitol and trehalose were also increased, with being accompanied by increased sucrose content. Furthermore, relative levels of carbohydrates, such as xylose, arabinose, glucose, sucrose, galactose and polyols were higher in XZ153 than in XZ141 (Fig. 4 and Table 1). XZ153 had the highest ribose and trehalose contents among three genotypes under LK stress, increasing by approximately 3.2 and 2.9 folds, respectively in comparison with the control (Table 1).

In contrast, LK stress caused a significant reduction of the metabolites involved in the TCA cycle, as shown by reduced contents of succinic acid, citrate, malic acid and ketoglutaric acid (Fig. 4 and Table 1), indicating that energy production through TCA cycle was deteriorated under LK stress. Interestingly there was a significant genotypic difference in the relative content of citrate, ketoglutaric acid, and malic acid under LK stress, with XZ141 showing more reduction than the other two genotypes (Fig. 4 and Table 1). In addition, contents of some organic acids, such as benzoic acid, maleic acid and ascorbic acid increased under LK stress, while quinic acid decreased in XZ141 (Fig. 4 and Table 1). Moreover, the genotypic difference was also detected in other metabolites, including urea, uracil and piperidine acids (Table 1).

### Metabolites profiles and pathway in barley leaves responding to LK stress

**Fig. 5:**
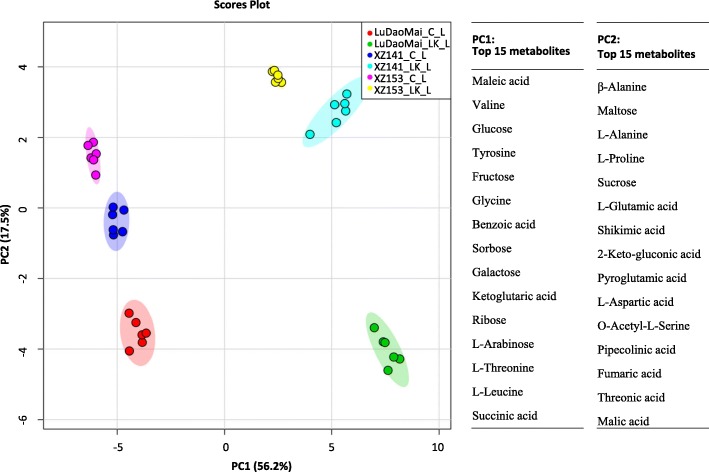
Leaf metabolome variation analysis and top 15 metabolites contributing to the PC1 and PC2. PC1: the first principal component; PC2: the second principal component; C: control; LK: low potassium; L: leaf (*n* = 6)

Leaf metabolomes of three genotypes were also dramatically changed under LK stress in comparison with the control (Table 2). Totally 52 metabolites altered significantly in their concentrations under LK stress compared to the control (Table 2). The results of PCA showed that PC1 could separate the samples of control and LK treatment, accounting for about 56.2% of the variation (Fig. 5 and Additional file 4: Table S4). Unlike in the roots, PC2 could clearly separate leaf samples within genotypes, explaining approximately 17.5% of the variation (Fig. 5). The major 15 metabolites contributing to the PC1 in leaves were 5 sugars (glucose, fructose, arabinose, sorbose and galactose) and 3 amino acids (valine, tyrosine and leucine), and 7 other metabolites, while the PC2 was dominated by fumaric acid, β-alanine, maltose, L-alanine and L-proline (Fig. 5 and Additional file 4: Table S4).

**Table 2:** Fold changes of detected metabolites in leaves of the three barley genotypes under LK stress

Metabolite name	Log_2_(LK/C)^a^			Metabolite name	Log_2_(LK/C)^a^		
	XZ153	XZ141	LuDaoMai		XZ153	XZ141	LuDaoMai
**Amino acid** ^b^				**Sugars and polyols**			
L-Alanine	0.01	0.51**	0.24**	Sucrose	0.06	0.13**	0.01
Valine	1.17**	1.39**	1.27**	Inositol	0.76**	0.30**	1.28**
L-Leucine	1.23**	1.84**	1.06**	**Organic acid**			
L-Isoleucine	1.01**	1.17**	1.65**	Glycolic acid	1.88**	1.29**	1.67**
L-Proline	0.78**	0.61**	0.71*	Benzoic acid	2.35**	1.59**	1.82**
Glycine	2.30**	3.41**	3.36**	Maleic acid	3.43**	2.79**	2.92**
L-Serine	1.00**	0.83**	0.80**	Succinic acid	−1.40**	−1.26**	−1.28**
L-Threonine	0.79**	0.81**	0.78**	Glyceric acid	−0.20*	−0.04	1.42**
β-Alanine	−0.96**	−0.52**	0.14	Fumaric acid	−0.35**	−0.50**	0.27**
L-Aspartic acid	−1.61**	−2.40**	−1.79**	Malic acid	−2.12**	−2.40**	−1.41**
GABA	0.13*	0.33**	0.05	Ketoglutaric acid	−3.59**	−4.73**	−2.51**
L-Glutamic acid	−1.69**	−2.25**	−1.21**	Threonic acid	−1.47**	− 1.09**	−1.83**
L-Phenylalanine	1.90**	1.38**	1.51**	Ribotide	−0.02	−0.95**	−0.03
L-Lysine	2.52**	1.82**	1.91**	Shikimic acid	−0.56**	−0.30*	0.92**
Tyrosine	2.30**	1.80**	2.15**	Citric acid	−4.77**	−4.99**	−3.58**
L-Asparagine	5.43**	3.98**	2.32**	Quinic acid	−0.17**	−0.30*	0.35**
L-Glutamine	3.78**	2.66**	2.43**	Ascorbic acid	0.12	−0.35*	0.69**
**Sugars and polyols**				Hexadecanoic acid	1.19**	1.38**	0.15*
Xylose	0.71**	1.05**	1.34**	Hexanoic acid	0.26	0.50**	−0.70**
L-Arabinose	1.85**	1.54**	1.74**	**others**			
Ribose	0.87**	2.14**	1.36**	Putrescine	5.30**	5.14**	4.54**
Xylitol	0.58**	0.59**	0.27*	Uracil	3.08**	1.35**	2.62**
2-Keto-L-gluconic acid	0.25*	0.27**	0.18*	Pipecolinic acid	3.83**	2.61**	0.71**
Fructose	2.83**	3.07**	3.43**	Threonic acid-1,4-Lactones	0.1	0.63**	1.02**
Galactose	3.75**	2.65**	2.81**	O-Acetyl-L-Serine	1.31**	0.55**	2.03**
Glucose	4.63**	3.57**	3.87**	Pyroglutamic acid	0.68**	−0.32**	−0.03
Sorbose	2.64**	2.63**	2.86**	O-Methyl-Inositol	0.91**	−0.04	0.12
Maltose	−0.50**	−0.52**	− 0.11	–	–	–	–

^a^The fold changes were calculated using the formula log_2_ (LK/C), *LK* Low potassium, *C* control. ^b^The bolder parts of the table are the different categories of metabolites.* and ** indicate significant (*p* < 0.05) and highly significant difference (*p* < 0.01), respectively

**Fig. 6:**
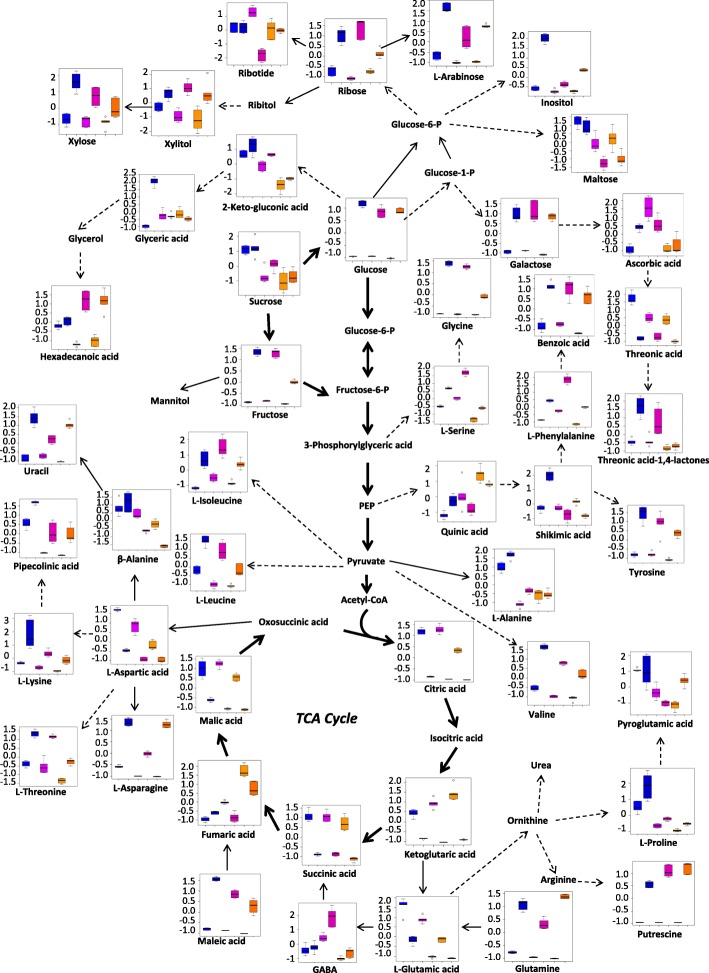
Metabolic pathways in leaves of the three barley genotypes in response to low-K stress. The six columns from left to right on the X-axis represent LuDaoMai (control and treatment), XZ141 (control and treatment) and XZ153 (control and treatment), respectively. The concentration of each metabolite on the Y-axis is presented after normalized on Metaboanalyst software (http://www.metaboanalyst.ca/)

Like the changes of root metabolites, the levels of more than two-thirds of amino acids were also enhanced in the leaves of the plants under LK-stress (Table 2). Among them, L-serine (about 2.0-fold), L-phenylalanine (3.7-fold), L-lysine (5.3-fold), tyrosine (4.9-fold), L-asparagine (43.1-fold) and glutamic acid (13.8-fold) had more increase in XZ153 than in XZ141 and LuDaoMai (Table 2). However, aspartic acid and glutamic acid were reduced in all three genotypes, with XZ141 being most affected (Fig. 6 and Table 2).

LK stress increased the contents of some soluble sugars (such as glucose, fructose and sucrose) in comparison with the control (Fig. 6 and Table 2). Hence, XZ153 showed the dramatic increase in the contents of glucose (approximately 24.8-fold), galactose (13.5-fold) and arabinose (3.6-fold) under LK stress, being much more increase than the other two genotypes. It is interesting to note that the effect of LK stress on sucrose content differed greatly between plant tissues and among genotypes. XZ153 and LuDaoMai had little change in leaves under LK stress, while showed a significant increase in roots (Fig. 6 and Table 2). In contrast, XZ141 showed the significant increase in leaves under LK stress, while little change in roots (Table 2).

In addition, TCA cycle was greatly inhibited under LK stress, as shown by lower contents of citric acid, ketoglutaric acid and succinic as well as other TCA intermediates compared to the control (Fig. 6 and Table 2). For instance, the contents of ketoglutaric acid and malic acid were significantly decreased in XZ141, being much lower than the other two genotypes. Moreover, reduced accumulation of ascorbic acid and pyroglutamic acid was recorded under LK stress in XZ141, but not in the other two genotypes (Table 2).

## Discussion

Potassium (K) is an essential inorganic cation for plant growth and development, and K deficiency is likely to lead to plant metabolic disturbances [31, 32]. It is well documented that there is an obvious difference among genotypes within a plant species in LK tolerance [7, 8, 33]. The current study confirmed the previous findings that the wild barley XZ153 is more tolerant to LK stress than XZ141 and LuDaoMai. The trend also reflected in the change of their metabolites when exposed to LK stress (Figs. 2, 3 and 5).

K is required as a counter cation for the electrical neutralization. Reduced K concentration in plant tissues and cells should lead to an electric charge imbalance [29, 32]. LK stress could result in the increase of some inorganic ions carrying positive charges (Na, Ca and Mg) in plant tissues [16, 32, 33], thus attributing to neutralization of negative charges. It was also reported that K deficiency in Arabidopsis altered metabolic charge in plant cells [29]. Armengaud et al. (2009) reported that the selective decrease of acidic amino acids could contribute to maintaining the charge balance in response to LK stress [29]. In this study, we also found that the synthesis of most amino acids was enhanced under LK stress (Fig. 4 and Tables 1 and 2). Moreover, the contents of two negatively charged amino acids, aspartic acid (Asp) and glutamic acid (Glu), were decreased in both leaves and roots (Tables 1 and 2), indicating that the alternation of these amino acid synthesis under LK stress could be associated with their function in adjusting charge balance. In fact, the amino acids carrying a positive charge, including lysine (Lys) and glutamine (Gln) increased in all three genotypes, with LK-tolerant XZ153 having more increase than the other two genotypes (Fig. 4 and Table 1). Meanwhile, in this study, we observed a new finding that the contents of organic acids in plant tissues were dramatically reduced under LK stress (Tables 1 and 2), which might be attributed to the requirement of reduced negative charge for charge balance in the LK-stressed plants. Therefore, it may be suggested that enhancement of positively charged amino acids and inhibition of negatively charged amino acids is a strategy for plants to keep charge balance in response to LK stress.

There are multiple secondary metabolic pathways in plants, and among them, the phenylpropanoid metabolic pathway is the most important [34]. L-phenylalanine, an aromatic amino acid derived from the shikimate pathway, is catalyzed by phenylalanine ammonia-lyase (PAL) into trans-cinnamic acid, a substrate for providing phenylpropanoid skeletons and it may further converted into flavonoids, lignin, alkaloids [35]. Thus, PAL is a key enzyme in the phenylpropanoid pathway [35]. It is well documented that PAL activity would be increased when plants are exposed to various biotic and abiotic stresses [36–39]. In addition, it was also reported that nitrogen and potassium deficiency increased PAL accumulation in plants [40, 41]. In our previous study, the results of proteomic analysis also showed that the protein level of PAL (M0XRA2 and F2DQ23) was enhanced in the LK-treated barley plants, and the tolerant genotype XZ153 had the highest increase [16]. Similarly, the results of this study showed that roots and leaves of XZ153 contained a higher content of L-phenylalanine than XZ141 and LuDaoMai under LK stress, thereby ensuring an adequate supply of substrate for PAL synthesis (Tables 1 and 2). Therefore, it may be concluded that secondary phenylpropanoid metabolic pathway mediated by PAL differs among barley genotypes, with higher LK tolerant genotype (XZ153) being most enhanced.

Carbohydrate metabolism plays central roles in the plant metabolism, providing energy for normal growth and development of plants, and acting as a bridge in the communications of protein, lipid and metabolism [42]. The increase in the contents of soluble sugars, including glucose, sucrose and fructose in plants is a typical response to different stresses [29, 43–46]. ACurrently, we found that the contents of most of the sugars in barley roots and leaves were significantly increased under LK stress, with LK-tolerant XZ153 having more increase than the other two genotypes (Figs. 4 and 6, Tables 1 and 2), indicating that more sugar accumulation should be one of the physiological traits for LK adaptation in plants, in views of the common functions of sugar and K in regulating osmotic potential. Correspondingly, XZ153 was least affected among the three genotypes in leaf chlorophyll content in response to LK stress (Fig. 1). On the other hand, the proteomic analysis showed that the proteins associated with photosynthesis, including photosystem I assembly protein Ycf4 (S4Z0P0), translocase of chloroplast 15 (M0Y503), magnesium-protoporphyrin IX (F2D7M5), CobN/magnesium chelatase (M0ZDL7) and mg-chelatase subunit XANTHA-G (F2CTR7), had more accumulation in XZ153 in comparison with other two genotypes [16]. The current results are basically consistent with those obtained in the proteomic study. Obviously, XZ153 showed relatively small change in the photosynthetic rate under LK stress, leading to less growth inhibition.

K-deficient is likely to be a consequence of impaired sucrose export from leaves, as loading of sucrose into the phloem is dependent on K [29, 32]. In this study, we found that sucrose was more accumulated in leaves but remained little or unchange in roots for XZ141, while the opposite was true for XZ153 and LuDaoMai (Figs. 4 and 6, Tables 1 and 2). Our previous study showed that XZ141 had the lowest K content in shoots under LK among the three barley genotypes [16]. Moreover, it was also reported that sucrose transported from leaf to root acts as a signal molecule involved in root growth regulation in response to nutrition deficiency [25, 47]. Coincidentally, we found that only in XZ141 RFW was significantly reduced under LK stress compared to its control (Fig. 1). Thus, it may be assumed that more sucrose accumulation of LK tolerant genotypes (XZ153) allows its better root growth under LK stress.

Hernandez et al. [48] reported that LK could cause an oxidative stress by producing excessive reactive oxygen species (ROS), resulting in destruction of the cell membrane stability. It is well documented that proline is an important antioxidant, and its accumulation in plant tissues is closely associated with stress tolerance [49]. In the present study, proline content in roots and leaves was increased under LK stress, with roots having more increase than leaves. Although the genotypic difference was not significant for proline increase in roots, XZ153 accumulated significantly higher proline content in leaves than XZ141 (Figs. 4 and 6, Tables 1 and 2). In addition, ascorbic acid is also an important scavenger of ROS in plants and has the function of protecting cell membrane permeability [50]. In this study, we found that the content of ascorbic acid differed greatly among genotypes, with XZ141 being much lower than XZ153 (Tables 1 and 2). Correspondingly, our previous study also showed that some proteins involved in detoxification of ROS were up-accumulated in the LK tolerant genotype XZ153 under LK stress, including cytochrome P450 superfamily protein (M0X9F3), NADPH-cytochrome P450 reductase (M0YD43) and ascorbate peroxidase (M0X2A1) [16]. Meanwhile, glucose-6-phosphate 1-dehydrogenase (G6PDH) (M0V4Z6), a key enzyme in the plant pentose phosphate pathway, also showed more increase in XZ153 than that in XZ141 and LuDaoMai, resulting in more NADPH for eliminating ROS [16, 51]. In addition, only LK sensitive genotype XZ141 showed the reduced three peroxidase proteins (M0WYK6, M0Y2V5, M0Z9D1) [16]. Obviously, XZ153 is highly capable of scavenging excessive ROS under LK condition, which should be associated with its higher adaptation to LK stress.

## Conclusions

**Fig. 7:**
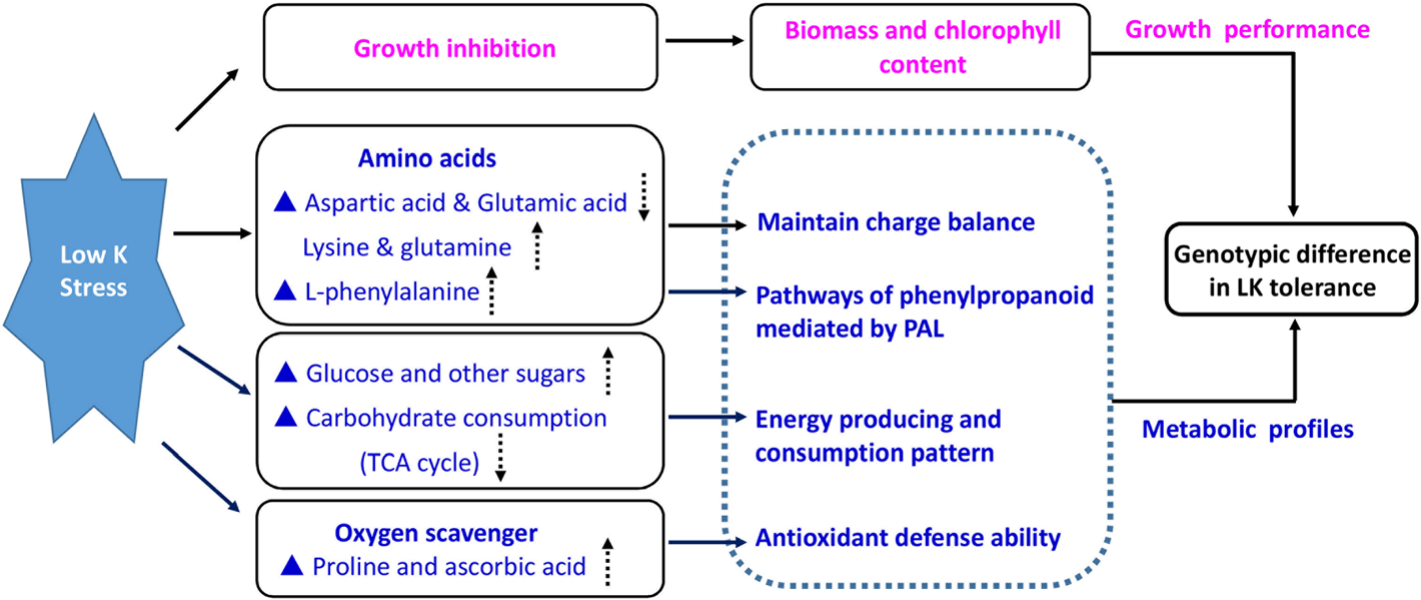
A hypothetically model of growth performance and metabolite profiles involved in the genotypic difference in low-potassium tolerance. The dotted arrows represent the content of metabolic was increased (up) and decreased (down) under low-K stress compared to the control, respectively

Based on the genotypic comparison in growth performance and metabolic profiles under control and LK treatment, a hypothetic model of genotypic difference in low-potassium adaptation could be presented (Fig. 7). It may be concluded that XZ153 is an LK-tolerant wild barley accession, which can be characterized by following traits under LK stress: (1) maintaining higher biomass and chlorophyll content; (2) enhancing phenylpropanoid metabolic pathway mediated by PAL; (3) saving energy by reducing carbohydrate consumption and storage of glucose and other sugars; and (4) developing a higher antioxidant defense ability. The metabolite profiles described here provide new insights into the ways how root and leaf metabolites are altered in barley under LK, and the mechanisms underlying genotypic difference in LK tolerance.

## Methods

### Barley materials and hydroponic culture

Two Tibetan wild barley accessions, XZ153 and XZ141 and a cultivated barley *cv*. LuDaoMai differing in low K (LK)-tolerant were used in the current study. Healthy seeds were sterilized with 2% H_2_O_2_ for 20 min and rinsed with distilled water for three times. Then, the seeds were germinated in a growth chamber (22/18 °C, day/night) in the dark. After the emergence of coleoptiles, the germination boxes were supplied with lights from fluorescent lamps. Ten-day-old seedlings with uniform size were selected and transplanted into plastic pots (5 L) for hydroponic culture. The experiment was conducted in a greenhouse with natural light. The hydroponic solution was prepared as described by Zeng et al. [16], and kept aerated during the whole process and renewed every five days.

### LK treatment and sampling

Three-leaf-old seedlings were initiated for LK treatment by adjusting the KCl concentration to 0.01 mM (low K). The seedlings grown in the solution containing 1 mM KCl were used as control. At the 16th day after K treatment, the plants were harvested from each treatment and control, and divided into shoots (include leaf blade and leaf sheath) and roots, then the fresh weight was recorded. The relative fresh weight was calculated as the ratio of each LK-treated plants to its respective control, with four biological replicates being set for each measurement. Meanwhile, roots and leaves (only leaf blade) were frozen in liquid nitrogen immediately and stored at − 80 °C for use in metabolite extraction.

### Metabolite extraction

Metabolite extraction in plant tissues was carried out according to Lisec et al. [52] with a small modification. The general workflow was as follows: root and leaf samples stored at − 80 °C were thoroughly ground with liquid nitrogen and the obtained powder (around 100 mg) was transferred into a 2 ml vertical centrifugal tube. Then the fine powder was extracted by adding 1.4 ml of 100% chromatogram methanol (pre-cooled at − 20 °C). The mixture was shaken for 15 s with vortex and then placed in a shaking water bath at 950 rpm for another 10 min at 70 °C, followed by centrifugation for 10 min at 11,000 g. The supernatant was transferred to a new 10 ml sterile centrifuge tube, and 750 μl chloroform (− 20 °C) and 1.5 ml deionized water (4 °C) was subsequently added, then mixed vigorously and centrifuged for 15 min at 2, 200 g. A total of 150 μl supernatant was dried in a vacuum freeze dryer for 60 min until the water disappeared completely. In the derivatization stage, 40 μl of 15 mg/ml methoxylamine hydrochloride pyridine solution was added into the dry samples, reacted for 2 h at 37 °C. Next, 70 μl of MSTFA reagent was added into the mixture, reacted for another 30 min. Finally, all the supernatant was transferred into glass vials suitable for GC-MS analysis. Meanwhile prepared one derivatization reaction was also prepared using an empty reaction tube as a control.

### GC-MS and metabolite analysis

The procedure for the GC-MS was described by Lisec et al. [52]. The injection, chromatography and mass spectrometer parameters were set accordingly. The mass spectra data were analyzed for resolution of peaks using AMDIS software [53]. The corresponding metabolites and their retention times were resolved by comparing with the known metabolites from commercial databases such as NIST (http://www.nist.gov/index.html). In addition, the initial content of the metabolite was calculated on the basis of the peak area. The Metabolic pathway was constructed by combining the KEGG metabolic database (http://www.genome.jp/kegg/) with the metabolic pathway summarized in the relevant references [54].

### Data analysis and statistical analysis

Before statistical analysis, the contents of all the metabolites were normalized using Metaboanalyst 3.0 online analysis software (http://www.metaboanalyst.ca/faces /ModuleView.xhtml). The identified metabolites were comprehensively compared by employing the principal component analysis (PCA) and the partial least squares-discriminant analysis (PLS-DA) methods in addition to the Heatmap analysis [55]. The fold-change of metabolite between control and treatment was transformed by logarithmic base of 2. The difference in the biomass, SPAD value and metabolite between control and treatment was tested using a data processing system (DPS) software. The difference at *p* < 0.05 and 0.01 are considered as significant and highly significant, respectively.

## Additional files

Additional file 1: Table S1. Concentrations of each metabolite in roots and leaves of the three barley genotypes under control and LK condition (XLSX 56 kb)

Additional file 2: Table S2. Loading factors contributing to PCA analysis of root metabolome variation (XLSX 11 kb)

Additional file 3: Table S3. Loading factors contributing to PLS-DA analysis of root metabolome variation (XLSX 10 kb)

Additional file 4: Table S4. Loading factors contributing to PCA analysis of leaf metabolome variation (XLSX 11 kb)

## Abbreviations

Ala: Alanine
Asn: Asparagine
Asp: Aspartic acid
C: Control
GABA: 4-aminobutyric acid
GC-MS: Gas chromatography-mass spectrometry
Gln: Glutamine
Glu: Glutamic acid
Gly: Glycine
K: Potassium
Leu: Leucine
LK: Low potassium
Lys: Lysine
PAL: Phenylalanine ammonia-lyase
PCA: Principal component analysis
Phe: Phenylalanine
PLS-DA: The partial least squares-discriminant analysis
RFW: Root fresh weight
Ser: Serine
SFW: Shoot fresh weight
Thr: Threonine
Tyr: Tyrosine
Val: Valine
